# The 5-HT and PLC Signaling Pathways Regulate the Secretion of IL-1*β*, TNF-*α* and BDNF from NG2 Cells

**DOI:** 10.1155/2022/7425538

**Published:** 2022-05-13

**Authors:** Tingting Yang, Yue Li, Hui Wang, Peng Shi, Liu Teng, Haibo Guo, Xiaohua Tu, Xinyu Yao

**Affiliations:** ^1^School of Basic Medicine, Guizhou University of Traditional Chinese Medicine, Guiyang, China; ^2^Graduate School, Chengde Medical College, Chengde, China

## Abstract

The present study was clarified the relationship between NG2 glial cells and 5-hydroxytryptamine (5-HT) to further revealed a role in the regulation of cortical excitability. The co-localization of NG2 cells and 5-HT in rat prefrontal cortex was determined using immunofluorescence. Different concentrations of 5-HT were applied to cultured NG2 cells. Real-time PCR measured the expression of interleukin-1*β* (IL-1*β*), tumor necrosis factor-*α* (TNF-*α*) and brain-derived neurotrophic factor (BDNF). Changes in the expression of IL-1*β*, TNF-*α*, and BDNF in NG2 cells were detected after the addition of 5-HT receptor specific blockers and phospholipase C (PLC) specific activators and inhibitors. The results confirmed that the NG2 protein and 5-HT co-localized in the prefrontal cortex. 5-HT treatment of NG2 cells significantly reduced the expression of IL-1*β* and BDNF mRNA and increased the expression of TNF-*α*. The 5-HT receptor specific inhibitors alverine citrate, ketanserin, ondansetron and SB-399885 blocked the regulatory effects of 5-HT on NG2 cells. The PLC signal was linked to the secretion of IL-1*β*, TNF-*α* and BDNF in NG2 cells. These results indicated that 5-HT affected IL-1*β*, TNF-*α*, and BDNF secretion from NG2 cells via the 5-HT1A, 5-HT2A, 5-HT3, 5-HT6 receptors and the PLC signaling pathway.

## 1. Introduction

NG2 glia cells are widespread cell populations throughout gray and white matter in the central nervous system (CNS) and have distinct morphological and physiological traits [1–3]. These cells specifically express NG2 chondroitin sulfate proteoglycan (CSPG4) and platelet-derived growth factor receptor alpha (PDGFR*α*) [4–6]. They are considered to be an independent population of glial cells, otherwise known as oligodendrocyte progenitor cells (OPCs) given that they can differentiate into oligodendrocytes during development [7, 8]. The formation of direct synaptic associations of NG2 cells with glutamatergic and *γ*-aminobutylergic neurons is a vital process in the transmission of information in the CNS [9] .Moreover, NG2 cells also secret some factors, IL-1*β*, TNF-*α* and BDNF, which affect the activity of CNS neurons and glial cells [10, 11]. Recent studies revealed that IL-1*β* and TNF-*α* influenced neuronal excitability and participated in immunoregulatory functions [12]. BDNF was implicated in the regulation of neuroplasticity, synaptic function, neuroprotection and excitation/inhibition imbalance [13].

5-HT is an important monoamine neurotransmitter in the CNS, and it is synthesized primarily in the raphe nuclei [14]. This neurotransmitter is a broad player in numerous processes, inducing neuronal excitability and modulating excitatory synaptic transmission and sleep-wake behavior [15, 16]. Serotonergic neurons fire most actively during wakefulness, reduce their rate of activity during non-rapid eye movement sleep (non-REMS), and are silent during rapid eye movement sleep (REMS) [17]. 5-HT affects the development, proliferation and differentiation of NG2 cells. It was recently reported that fluoxetine increased 5-HT inhibits the basal proliferation and survival of OPCs in the fornix and corpus callosum of adult mice [18]. PLC is an enzyme that mediates cell signaling through various metabolic receptors. Among several PLC subtypes, the *β*4 subtype is uniquely localized in the geniculate nucleus of the thalamus, which is postulated to have a key role in the transition and maintenance of sleep stages. PLC-*β*4−/− mice exhibited increased REM sleep and unusual wake-to-REM sleep transitions at night [19]. The actions of the 5-HT (2A), 5-HT (2B) and 5-HT (2C) receptor subtypes are mediated by the activation of PLC, resulting in depolarization of the host cell [16]. Our previous study revealed that the down-regulation of 5-HT synthesis via parachlorophenylalanine (PCPA) produced NG2 cell activation and proliferation. Other functional relationships between 5-HT and NG2 cells may exist, and further studies should investigate the relevant mechanisms of 5-HT.

The present study examined whether NG2 cell secretion of IL-1*β*, TNF-*α* and BDNF was associated with 5-HT. The results showed that the effects of 5-HT on NG2 cell secretion was related to 5-HT1A, 5-HT2A, 5-HT3 and 5-HT6 receptors and the PLC signaling pathway.

## 2. Materials and Methods

### 2.1. Experimental Animals

Adult male Sprague-Dawley (SD) rats (Tianqin Biotechnology Co. Ltd. China) were used for immunofluorescence study. All relevant experiments were performed in accordance with the protocol of the National Institutes of Health and institutional guidelines for the humane care of animals. All efforts were made to reduce the number of animals used and minimize any pain and discomfort.

### 2.2. Cell Culture and Treatment

NG2 cells, namely rat oligodendrocyte precursor cells (ROPCs), were provided by Qingqi (Shanghai, China) Biotechnology Development Co, Ltd. Cells were routinely cultivated in dulbecco's modified eagle medium (DMEM) with high glucose (Gibco, USA) supplemented with 10% fetal bovine serum (FBS), penicillin (100 U/ml) and streptomycin (100 µg/ml) at 37°C in a 5% CO_2_ humidified atmosphere.

For 5-HT-induced NG2 cell secretion assays, according to the results of the pre-experiment, the cells were treated with 5-HT (25, 50 and 100 *μ*M) for 24 hours. NG2 cells were treated with various receptor (5-HT) inhibitors and specific activators/inhibitors of intracellular signals, such as 5-H1A, 5-HT2A, 5-HT3 and 5-HT6 receptors, including the 5-HT1AR, 5-HT2AR and 5-HT6R nonspecific antagonist asenapine maleate (Ase, 2.5 *μ*M), the 5-HT1A specific receptor inhibitor alverine citrate (1.25 *μ*M), the 5-HT2A specific receptor inhibitor ketanserin (37.5 *μ*M), the 5-HT3 specific receptor inhibitor ondansetron (5 *μ*M), the 5-HT6 specific receptor inhibitor SB-399885 (1 *μ*M), the PLC specific activator m-3M3FBS (3 *μ*M) and the specific inhibitor U-73122 (2.5 *μ*M). These reagents were applied with 5-HT (100 *μ*M) for 24 hours.

### 2.3. Immunofluorescence Double-Labeling

The brain was removed after perfusion and the prefrontal cortex was cut into 30-*μ*M sections. Sections were blocked with 10% goat serum for 30 minutes at 37°C, and the liquid was aspirated without washing, and then incubated with primary antibodies for 2 hours at 37°C protected from light. The primary antibodies included anti-NG2 monoclonal antibodies (NG2-Ab, 1 : 80, Abcam, USA) and anti-TPH (TPH-Ab, 1 : 200, Abcam, USA). Subsequently, sections were incubated with secondary antibodies, including ALexa Fluor 488 (green, 1 : 100) and ALexa Fluor 594 (red, 1 : 100, both from Invitrogen, USA), for 1 hour at 37°C protected from light. These were followed by counterstained with a DAPI Mix (Beijing Solebro Technology Co, China). Finally, sections were observed under a fluorescence inverted microscope (Olympus, Japan). Fluorescence was detected using excitation wavelengths of 488 nm (green), 649 nm (red) and 358 nm (blue) , respectively. NG2 cells (red fluorescence) and 5-HT neuronal markers (green fluorescence) were observed. When two immunofluorescence images overlapped in yellow (red + green = yellow), the two markers exhibited co-expression [20].

### 2.4. ELISA Analysis

NG2 cells in the exponential growth phase (2 × 10^5^ cells/well) were seeded in 6-well plates and treated with each group for 24 hours. The protein levels of TNF-*α*, IL-1*β* and BDNF in the cell supernatants were detected using ELISA kits, according to the manufacturer's instructions. ELISA kits for rat IL-1*β* (Shenzhen Xin Bosheng Biotechnology Co, China), TNF-*α* (Shenzhen Xin Bosheng Biotechnology Co, China) and BDNF (Wuhan Elixirite Corporation, China) [21].

### 2.5. Real-Time PCR Analysis

NG2 cells in the exponential growth phase (2 × 10^5^ cells/well) were seeded in 6-well plates and treated with each group for 24  hours. Intracellular total RNA was extracted using a kit (Feiyang Biological Engineering Co, Guangzhou, China). cDNA was reverse transcribed with a kit (Feiyang Biological Engineering Co, Guangzhou, China), and 2 × SYBR Green qPCR Mix (Yisun Biotechnology Co, Shanghai, China) was used for real-time PCR. Real-time PCR (StepOne Plus, ABI Corporation, USA). Glyceraldehyde-3-phosphate dehydrogenase (GAPDH) was used as the internal reference gene. The amplified products were resolved by 1.5% agarose gel electrophoresis and visualized using ethidium bromide staining and a UV light source. Each experiment was repeated three times with three replicate wells, and the results were analyzed using the 2^−ΔΔCt^ method to determine the relative expression. All primers and the related sequences used in the experiments are shown in Table 1.

### 2.6. Western Blot Analysis

NG2 cells in the exponential growth phase (2 × 10^5^ cells/well) were seeded in 6-well plates, and all the experimental groups were incubated for 24  hours. The protein expression of PLC (Abclonal, China), IL-1*β* (Cell Signaling, China), TNF-*α* (Cell Signaling, China) and BDNF (Bioss, China) was determined using Western blot (WB) analysis. Protein extracts were separated using 5% gum concentrate and 12% separating adhesive and transferred to PVDF membranes. The membranes were blocked with TBST buffer and kept at room temperature for 2 hours on a shaker. The corresponding primary antibody was diluted in the blocking solution and the PVDF membranes were immersed in the primary antibody incubation solution overnight at 4°C. The membranes were rinsed three times with TBST buffer and incubated with a secondary Ab for 2 hours at room temperature. After four rinses with TBST buffer, the PVDF membranes were scanned and imaged.

### 2.7. Statistical Analysis

SPSS software was used for statistical analysis (version 25.0). Outcomes are presented as the means ± SD from at least three separate experiments. Distinctions between groups were analyzed using one-way analysis of variance (one-way ANOVA) followed by post hoc tests using LSD or Dunnett's test. Statistical differences at ^*∗*^*p* < 0.05 were deemed significant, and ^*∗∗*^*p* < 0.01 was considered extremely significant.

## 3. Results

### 3.1. Colocalization of NG2 Cells with 5-HT Neuronal Markers (TPH) in the Prefrontal Cortex

**Figure 1:**
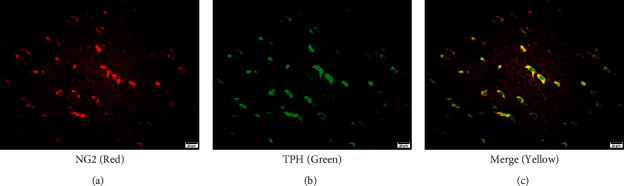
Colocalization of NG2 cells and 5-HT neuron markers in the rat prefrontal cortex. Double fluorescence detection of NG2 cells (red; (a)) and the 5-HT neuron marker TPH (green; (b)) in the prefrontal cortex. The merged images show colocalization of NG2 cells and 5-HT neurons in the prefrontal cortex (c). Scale bar = 20 *μ*m.

To determine whether NG2 cells co-localized with 5-HT neuronal markers (TPHs) in the prefrontal cortex, double immunofluorescence staining was performed using NG2 and TPH antibodies. As shown in Figure 1, NG2 cell signals were identified as green fluorescence (Figure 1(a)), and 5-HT neuron marker (TPH) signals were recognized as red fluorescence (Figure 1(b)). The yellow color in double fluorescence detection showed that nearly all 5-HT neuron markers (TPH) co-labeled with NG2 cells in the prefrontal cortex (Figure 1(c)). These results suggest a functional relationship between NG2 cells and 5-HT.

### 3.2. Expression of 5-HT3, 5-HT5, 5-HT6, 5-HT7 Receptors and 5-HT1A, 5-HT2A Subtypes on NG2 Cells

**Figure 2:**
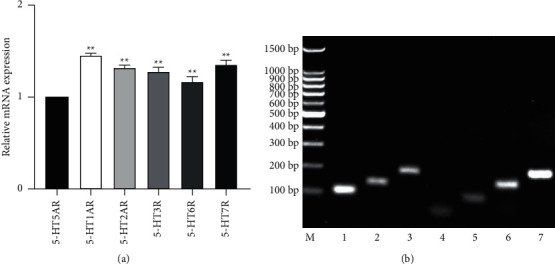
5-HTRs expressed on NG2 cells. (a) Data presented as expression relative to 5-HT5. (Data are expressed as the means +/− s.d: *n* = 3 for each group. ^*∗*^*p* < 0.05, ^*∗∗*^*p* < 0.01. s.d: standard deviation). (b) The PCR products were visualized in agarose gels. Comparison of the PCR products was performed using GAPDH as a positive control. Controls are labeled as follows: M, DNA marker; 1, 5-HT1AR; 2, 5-HT2AR; 3, 5-HT3R; 4, 5-HT5R; 5, 5-HT6R; 6, 5-HT7R; 7, GAPDH. The product size of each receptor matched the expected product size.

To further investigate the functional relationship between NG2 cells and 5-HT, real-time PCR was used to detect 5-HT receptors expressed on NG2 cells. The expression of the 5-HT1A, 5-HT2A, 5-HT3, 5-HT5, 5-HT6 and 5-HT7 receptors was detected on NG2 cells (Figure 2).

### 3.3. 5-HT Induced IL-1*β*, TNF-*α* and BDNF Secretion from NG2 Cells

**Figure 3:**
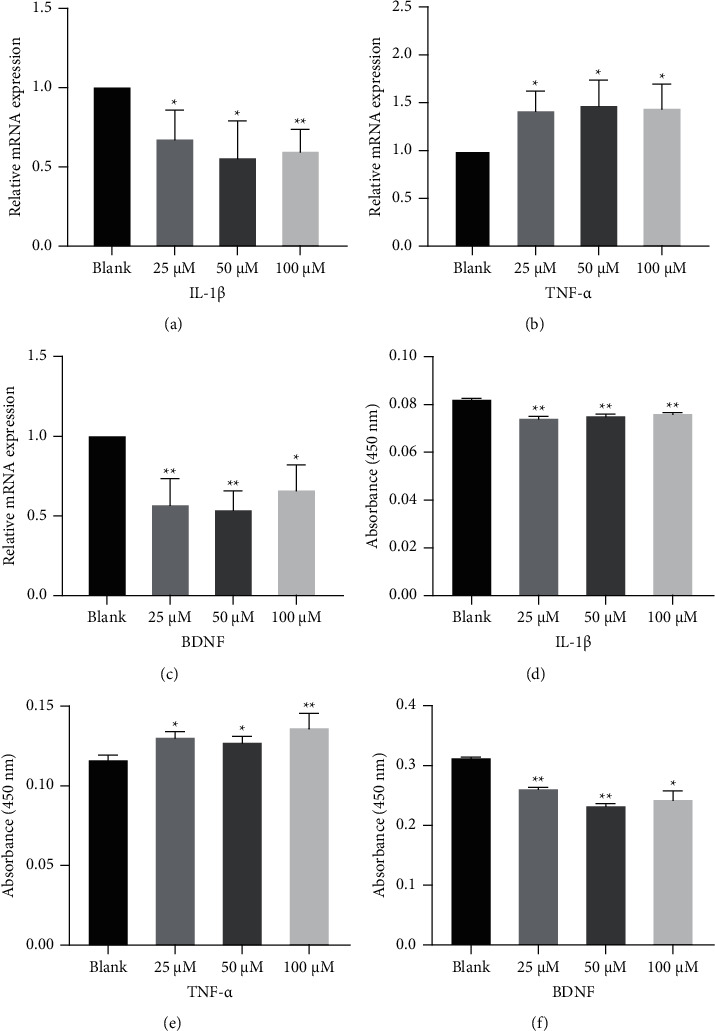
5-HT treatment induces IL-1*β*, TNF-*α* and BDNF secretion in NG2 cells. (a-c) IL-1*β*, TNF-*α* and BDNF mRNA levels were determined using real-time PCR. (d-f) IL-1*β*, TNF-*α* and BDNF protein expression levels were measured using ELISA. (Data are expressed as the means +/− s.d. n = 3 for each group. ^*∗*^*p* < 0.05, ^*∗∗*^*p* < 0.01. s.d.: standard deviation).

To investigate whether the secretion of IL-1*β*, TNF-*α* and BDNF in NG2 cells was associated with 5-HT, we used different concentrations of 5-HT (25, 50, and 100 *μ*M) to stimulate NG2 cells. Figure 3 shows that 5-HT significantly inhibited the secretion of IL-1*β* and BDNF and significantly increased TNF-*α* secretion. These results showed that 5-HT induced the secretion of IL-1*β*, TNF-*α* and BDNF in NG2 cells.

### 3.4. 5-HT Modulates NG2 Cells Secretion of IL-1*β*, TNF-*α* and BDNF via 5-HT1AR, 5-HT2AR, 5-HT3R and 5-HT6R

**Figure 4:**
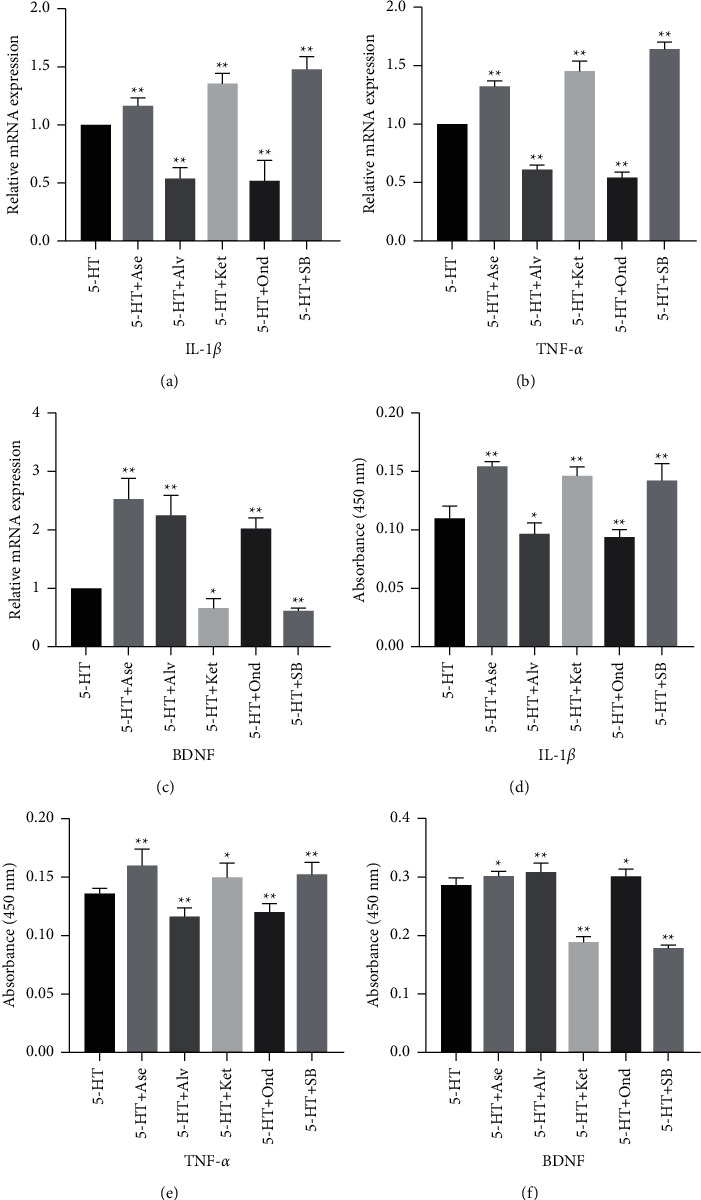
Effects of various antagonists of 5-HTRs on IL-1*β*, TNF-*α* and BDNF secretion from NG2 cells. (a-c) IL-1*β*, TNF-*α* and BDNF mRNA levels were estimated using real-time PCR. (d-f) IL-1*β*, TNF-*α* and BDNF protein expression was measured using ELISA. (Data are expressed as the means +/− s.d. n = 3 for each group. ^*∗*^*p* < 0.05, ^*∗∗*^*p* < 0.01. s.d.: standard deviation).

There are multiple 5-HT receptor types on NG2 cells. Therefore, we investigated the receptors involved in IL-1*β*, TNF-*α* and BDNF secretion. We used the nonspecific antagonist asenapine maleate (Ase) to block 5-HT1AR, 5-HT2AR and 5-HT6R and found elevated secretion of IL-1*β*, TNF-*α* and BDNF in NG2 cells (Figure 4). We used specific antagonists alverine citrate (Alv), ketanserin (Ket), ondansetron (Ond) and SB-399885 (SB), to block 5-HT1AR, 5-HT2AR, 5-HT3R and 5-HT6R, respectively. Blockade of 5-HT1AR or 5-HT3R down-regulated the secretion of IL-1*β* and TNF-*α* and elevated BDNF secretion (Figure 4). Blockade of 5-HT2AR and 5-HT6R reduced the secretion of BDNF and increased the secretion of IL-1*β* and TNF-*α* (Figure 4). These results indicated that 5-HT modulated IL-1*β* and TNF-*α* secretion via 5-HT1AR and 5-HT3R in NG2 cells. 5-HT modulated the secretion of BDNF via 5-HT2AR and 5-HT6R.

### 3.5. Role of PLC in the Secretion of Il-1*β*, TNF-*α* and BDNF in NG2 Cells

**Figure 5:**
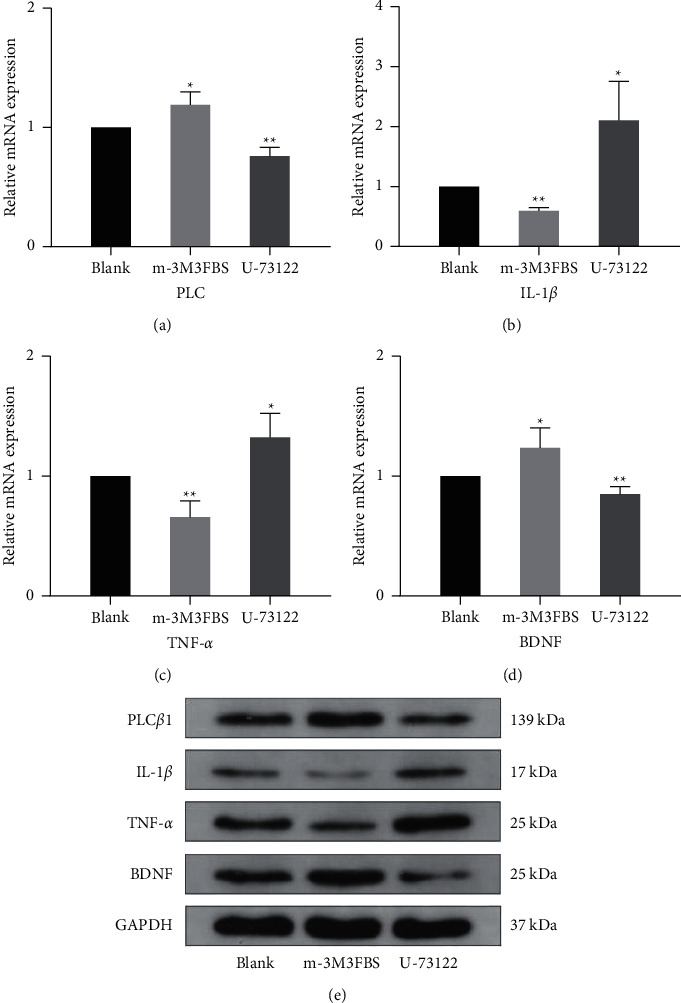
Effects of the specific activator m-3M3FBS and the specific inhibitor U-73122 of PLC on the secretion of IL-1*β*, TNF-*α* and BDNF in NG2 cells. (a-d) Real-time PCR was used to determine the expression of PLC, IL-1*β*, TNF-*α* and BDNF mRNA. (d-e) Western blotting was used to determine the expression of PLC, IL-1*β*, TNF-*α* and BDNF. GAPDH is shown as an internal control. (Data are expressed as the means +/− s.d. n = 3 for each group. ^*∗*^*p* < 0.05, ^*∗∗*^*p* < 0.01. s.d.: standard deviation).

The effects of PLC on NG2 cell secretion of IL-1*β*, TNF-*α* and BDNF were determined using the PLC-specific activator m-3M3FBS and the specific inhibitor U-73122. As indicated in Figure 5, m-3M3FBS increased the expression of PLC in NG2 cells and increased the secretory level of BDNF but decreased IL-1*β* and TNF-*α* secretion from NG2 cells (Fig.  5(a)–5(d)). U-73122 decreased the expression of PLC and the secretion of BDNF but increased the levels of IL-1*β* and TNF-*α* in NG2 cells (Fig.  5(a)–5(d)). These results were confirmed using Western blotting (Figure 5(e)). These data demonstrated that secretion of IL-1*β*, TNF-*α* and BDNF in NG2 cells was associated with PLC signaling.

## 4. Discussion

With the widest distribution and the largest number of neural cells in the central nervous system, glial cells fulfil various physiological functions, including the orchestration of synaptic activity, maintenance of ion homeostasis, ensuring the integrity of the blood–brain barrier, and participating in neurotransmitter uptake [22, 23]. Previous studies confirmed the role of glial cells in the regulation of non-rapid eye movement sleep and cognitive functions [24]. Glial cells orchestrate the sleep-wake cycle by releasing different neurotransmitters, such as glutamate, serine, and adenosine triphosphate (ATP), and secrete pro-inflammatory cytokines, such as IL-1*β* and TNF-*α*, which aid in sleep. Glia also participate in the regulation of sleep homeostasis by regulating the level of extracellular adenosine and the activity of adenosine receptor-1 (A1R) or adenosine receptor 2A (A2AR) on different neuronal membranes [25–27]. The number of NG2 cells increases during sleep increases compared to arousal and during sleep deprivation [28].The proliferation of NG2 cells is positively related to the frequency of rapid eye movement during sleep. The occurrence of this effect is orchestrated by Glu signal transduction initiated by neurons. This relationship suggests that the excitability of the central nervous system influences the proliferation and differentiation of NG2 cells [29, 30]. Activated NG2 cells secrete substances that affect the activity of neurons and glial cells [31, 32]. Ablation of NG2 cells in the prefrontal cortex adversely affects Glu receptors and Glu neurotransmission and disrupts Glu-related astrocytes and neuronal functions, which affect the excitability of the central nervous system.

In summary, NG2 cells may be related to central nervous system excitability and sleep-wake regulation. Notably, IL-1*β*, TNF-*α* and BDNF are associated with cortical excitability and sleep-wake. Increased expression of IL-1*β* and TNF-*α* in the brain was detected in rats completely deprived of sleep for 24 hours [33]. High levels of exogenous IL-1*β* and TNF-*α* expression induce slow-wave sleep [34]. Increased expression of BDNF was detected in the hippocampus of mice after 1 day of sleep deprivation, and BDNF expression declined after 3 days of sleep deprivation [11]. These studies suggest that the roles of IL-1*β*, TNF-*α* and BDNF in sleep regulation are related to the degree of insomnia and the concentration and duration of IL-1*β*, TNF-*α* and BDNF effects. This study found that different concentrations of 5-HT (25, 50, 100 *μ*M) stimulated NG2 cells to secrete IL-1*β*, TNF-*α* and BDNF. 5-HT (25, 50, 100 *μ*M) significantly inhibited the secretion of IL-1*β* and BDNF, while 5-HT (25, 50, 100 *μ*M) significantly increased the secretion of TNF-*α*. The results showed that 5-HT could significantly increase the secretion of TNF-*α*. NG2 cells were induced to secrete IL-1*β*, TNF-*α* and BDNF.

Our study revealed that 5-HT acted on NG2 cells to regulate the secretory levels of IL-1*β*, TNF-*α* and BDNF. 5-HT may regulate the release of IL-1*β*, TNF-*α*, BDNF and other sleep regulation-related active substances by affecting the glial system, which regulates the neuronal and glial cell activity involved in the regulation of cortical excitability. Their specific roles and mechanisms are discussed further below.

The raphe nucleus group, located in the reticular structure of the brainstem, contains the cell bodies of 5-HT neurons [35, 36]. Destruction of the raphe nucleus using electrocoagulation or serotonin receptor antagonists causes insomnia in animals. The use of p-chlorophenylalanine to deplete 5-HT in the brain also causes severe insomnia depending on the animal. This insomnia may be recovered by intracerebral injections of 5-HT. The injection of 5-HT into the fourth ventricle of a lightly anesthetized cat causes sleep-like brain waves [37, 38]. The RNA and protein of glial cells and neurons in the dorsal raphe nucleus of rats with sleep deprivation were found decreased 23% and 31%, respectively. After 48 hours, the RNA content reached normal levels, but the protein content was 21% lower than normal. Natural sleep increased the RNA concentration and absolute content of glial cells in locus coeruleus nucleus but reduced the absolute content of neuronal cytoplasmic protein. The protein concentration of neurons and glial cells increased, and the absolute content of glial cell protein increased. The concentration and absolute content of RNA were the same as the control group. Sleep regulation is closely related to 5-HT and glial cells, which affect cortical excitability [39, 40]. The present study explained, for the first time, the regulatory relationship between NG2 cells and 5-HT, which is consistent with previous studies on glial cells.

5-HT acts via 5-HT receptors. The 5-HTRs in mammals are organized into seven families (5-HT1-7R), and more than 16 subtypes have been described. 5-HT1AR and 5-HT1BR activation may hyperpolarize the cell membrane by inhibiting adenylate cyclase. 5-HT2AR and 5-HT2BR regulation by PLC leads to cell membrane depolarization. 5-HT3R activation depolarizes acetylcholine and amino acidergic neurons, and 5-HT6R and 5-HT7R receptor activation leads to depolarization of neurons that participate in the regulation of cortical excitability [41]. 5-HT functions as an activator in waking (W) but hinders REMS. The situation is similar in cats and rodents, and the process may be accomplished via 5-HT1R. 5-HT1AR and 5-HT1BR reduce REM sleep in mice, and 5-HT2AR, 5-HT2CR or 5-HT7R produce the opposite effect [42]. These studies suggest that 5-HT, acting on its receptors, is involved in cortical excitability that affects sleep regulation. PLC mediates cellular signaling via various metabotropic receptors. 5-HT2AR couples with the G*α*q, G*α*i/*o* and G*α*12/13 proteins in vitro and in vivo. Stimulation of the G*α*q pathway activates PLC, generates inositol-1,4,5-triphosphate (IP3) and diacylglycerol (DAG), and releases calcium from intracellular stores to induce specific biological effects. The present study used 5-HT2AR receptor inhibitors and PLC signaling pathway inhibitors and showed consistent trends in the secretory levels of IL-1*β*, TNF-*α* and BDNF from NG2 cells. Our results suggest that 5-HT regulates the secretion of IL-1*β*, TNF-*α* and BDNF by affecting the 5-HT2AR-coupled PLC signaling pathway in NG2 cells. In summary, 5-HT may regulate cytokine activity via interaction with NG2 cells, which affects cortical excitability.

## 5. Conclusion

NG2 positive cells and 5-HT neuronal marker TPH were co-localized in the prefrontal cortex. 5-HT1A, 5-HT2A, 5-HT3, 5-HT6, and 5-HT7 receptors can be expressed on NG2 cells. 5-HT affected NG2 cells secretion of IL-1*β*, TNF-*α*, and BDNF via 5-HT1A, 5-HT2A, 5-HT3, and 5-HT6 receptors, through PLC signaling pathway.

## Figures and Tables

**Table 1:** Primer sequences used in this study.

Primer name		Sequences (5ʹ-3ʹ)	Product size (bp)
5-HT1AR	Forward	AAGGACCACGGCTACACCATCTAC	108
	Reverse	CTGACAGTCTTGCGGATTCGGAAG	
5-HT2AR	Forward	CTTCCAACGGTCCATCCACA	132
	Reverse	GGGCACCACATTACAACAAACAG	
5-HT3R	Forward	ACATTTCCCTGTGGCGAACA	189
	Reverse	CAGTGGTTTCCCATGGCTGAG	
5-HT5R	Forward	CGCTGTGCTCCTGGGATAT	78
	Reverse	CCTGTTGAACGCCGTGTAGAT	
5-HT6R	Forward	GCACGAACTGGGCAAAGCT	86
	Reverse	GGACGCCACGAGGACAAAA	
5-HT7R	Forward	TTCTGTCGGTCTGGCTGCTCTC	130
	Reverse	CCGCAGTGGAGTAGATCGTGTAG	
IL-1*β*	Forward	TGACAGGCAACCACTTACC	123
	Reverse	CCCATACACACGGACAACT	
TNF-*α*	Forward	GAAACAGTCTGCGAGGTGTG	158
	Reverse	TTCTTCTTGCAGCCACACAC	
BDNF	Forward	TCTACGAGACCAAGTGTAATCCCAT	166
	Reverse	GAAGTGTCTAT CCTTATGAACCGC	
PLC*β*1	Forward	CTGAGGGCTCACGCAAGAA	102
	Reverse	GCAGCACGGTATAGGTGAA	
GAPDH	Forward	ACAGCAACAGGGTGGTGGAC	98
	Reverse	TTTGAGGGTGCAGCGAACTTT	

## Data Availability

The data used to support the findings of the study are available from the corresponding author upon reasonable request.
